# Molecular insights into immune evasion in head and neck squamous cell carcinomas: Toward a promising treatment strategy

**DOI:** 10.32604/or.2025.062207

**Published:** 2025-05-29

**Authors:** HYEON JI KIM, BO KYUNG JOO, JIN-SEOK BYUN, DO-YEON KIM

**Affiliations:** 1 Department of Pharmacology, School of Dentistry, Kyungpook National University, Daegu, 41940, Republic of Korea; 2 Department of Oral Medicine, School of Dentistry, Kyungpook National University, Daegu, 41940, Republic of Korea; 3 Brain Science and Engineering Institute, Kyungpook National University, Daegu, 41940, Republic of Korea

**Keywords:** HNSCC, Human papillomavirus, Tumor microenvironment, Tumor-associated macrophage, Cancer-associated fibroblast

## Abstract

Head and neck squamous cell carcinoma (HNSCC) is a highly aggressive and devastating disease arising primarily from the mucosal epithelium of the oral cavity, pharynx, and larynx. HNSCC ranks as the sixth most common cancer worldwide, carrying significant morbidity and mortality. HPV-positive HNSCC can be partially prevented with the FDA-approved HPV vaccine and generally exhibits a more favorable prognosis compared to HPV-negative cases. However, effective screening and treatment approaches remain elusive for HPV-negative HNSCC. While precancerous lesions may precede invasive cancer in certain situations, most patients present with advanced disease without prior indication of precancerous conditions. Despite robust immune cell infiltration in HNSCC tumors, the extent and composition of immune infiltration vary widely among patients, and these tumors often evade immune surveillance through diverse mechanisms. Given the heterogeneous nature of HNSCC influenced by anatomical location and etiological factors, precise identification of biomarkers and personalized treatment strategies are imperative. In this study, we aim to explore the possibility of establishing an effective treatment strategy to overcome obstacles to targeted treatment and enable long-term survival through detailed molecular characterization and immune profiling of HNSCC.

## Introduction

Head and neck squamous cell carcinoma (HNSCC) is the sixth most common malignancy worldwide and is associated with a poor prognosis [1]. Treatment for HNSCC typically involves a combination of radiation therapy, surgery, and chemotherapy. Despite these aggressive approaches, survival rates remain suboptimal, highlighting the need for innovative treatment strategies. Recently, immune checkpoint inhibitors, particularly anti-PD-1/PD-L1 agents such as nivolumab and pembrolizumab, have shown promise in reactivating the immune system to target and eliminate tumor cells of HNSCC [2,3]. However, anti-PD-1/PD-L1 agents exhibit a low response rate of approximately 20% in HNSCC patients [4]. This limited efficacy is often attributed to complex immunosuppressive mechanisms that enable tumor cells to evade immune surveillance, thereby restricting the effectiveness of immunotherapy [5,6].

Cancer cells create an immunosuppressive microenvironment that impairs both innate and adaptive immune responses, partly through the secretion of immunosuppressive cytokines such as interleukin-10 (IL-10) and transforming growth factor beta (TGF-β) [7].

A comprehensive understanding of the molecular mechanisms underlying immune evasion is essential to enhance the efficacy of immunotherapy in HNSCC. This study aims to uncover critical pathways that facilitate immune evasion in HNSCC, with the objective of identifying novel therapeutic targets and strategies. Such insights could ultimately pave the way for improving treatment outcomes and patient survival.

## Differences between HPV-Positive and HPV-Negative HNSCC

### The cancer immune landscape in response to HPV infection

HNSCC can develop as a result of malignant transformation following infection with high-risk human papillomavirus (HPV) [1]. Beyond initiating tumorigenesis, HPV infection significantly reconfigures the tumor microenvironment (TME), shaping the cancer immune landscape. Regardless of the location of origin, HPV-positive tumors are characterized by a higher density of immune cells, including B cells, T cells, and natural killer (NK) cells. In contrast, the TME of HPV-negative tumors is predominantly enriched with cancer-associated fibroblasts (CAFs), dendritic cells (DCs), endothelial cells, perivascular cells, and myofibroblasts [8]. This immunologically rich environment in HPV-positive cases correlates with a favorable survival outcome compared to HPV-negative patients [9].

Insight into the immune landscape of HPV-positive and HPV-negative HNSCC patients is important to optimize immunotherapeutic approaches.

In general, the TME contains various immune cells, including regulatory T cells (Tregs), CD8+ T cells, CD4+ T cells, and B cells. Both subtypes are characterized Tregs, but HPV-positive HNSCC are present in lower proportions compared to HPV-negative patients [10]. CD8+ T cells are also observed in both subtypes, their proportion is significantly higher in HPV-positive tumors (27.1% vs. 14.7%) [11]. Macrophage infiltration was found to be higher in HPV-positive HNSCC compared to HPV-negative tumors, with a higher infiltration of M1 subtype macrophages expressing immune activation phenotypes [12]. Plasmacytoid dendritic cells (pDCs) do not differ between the two subtypes, whereas the proportion of functionally intact pDCs is higher in HPV-positive HNSCC [13]. Additionally, natural killer (NK) cells are more abundant in HPV-positive tumors compared to HPV-negative tumors (82% vs. 57%) [14]. In contrast, the predominance of M2 subtype macrophages in HPV-negative tumors promotes tumor progression and correlates with poorer prognosis [15]. In addition, mast cells are extremely abundant in the HPV-negative tumor microenvironment, contributing to immunosuppressive signaling [16]. Tumor-associated neutrophils (TANs) are known to have a higher infiltration rate in HPV-negative HNSCC compared to HPV-positive tumors [17]. Myeloid-derived suppressor cells (MDSCs) are significantly distributed in HPV-negative HNSCC, but further studies are necessary to ascertain the proportion of MDSCs in patients with HNSCC based on their HPV status [18].

### Potential biomarkers for immune evasion

HPV infection activates the PI3K/MAPK/AKT/mTOR pathway in a variety of ways, contributing to the generally better prognosis for HPV-positive patients compared to HPV-negative patients, largely due to frequent infiltration of Tregs and NK cells [19,20]. While the PI3K/AKT/mTOR pathway can enhance T cell and NK cell function, excessive activation leads to immune cell exhaustion and suppression, ultimately impairing an effective anti-tumor response [21,22]. Despite these differences, therapeutic strategies for patients with HNSCC remain largely uniform, irrespective of HPV status [23].

In HPV-positive HNSCC, Toll-like receptor (TLR) and T-cell receptor signaling, PD-L1/PD-1 checkpoint activation, and the NF-kappa B (NF-κB) pathway play key roles in modulating the immune environment [24]. In addition, HPV-positive tumors exhibit higher expression of immune checkpoint genes, including cytotoxic T-lymphocyte associated protein 4 (CTLA4), lymphocyte-activation gene 3 (LAG3), PD1 gene (PDCD1), T-cell immunoglobulin domain and mucin domain-3 (HAVCR2), programmed Cell Death 1 Ligand 2 (PDCD1LG2), and T cell immunoreceptor with immunoglobulin and ITIM domain (TIGIT), which likely contribute to enhanced immune evasion [25].

In HPV-positive HNSCC, biomarkers such as Integrin alpha 5 (ITGA5), Transforming growth factor beta 1 (TGFB1), Plasminogen Activator, Urokinase (PLAU), and Plasminogen activator inhibitor-1 (SERPINE1) are associated with immune cell infiltration and increased radiosensitivity through NF-κB activation [26]. In addition, follicular dendritic cell secretory protein (FDCSP) expression shows positive correlations with B cells, CD4+ memory resting cells, Tregs, and M1 macrophages [27]. Dedicator of Cytokinesis 8 (DOCK8) regulates lymphocyte function by inducing both innate and adaptive immune responses via interleukin-2 (IL-2) and signal transducer and activator of transcription 5 (STAT5) pathways, alleviating immunological tolerance and tumorigenesis [28]. Additionally, Cannabinoid receptor 2 (CNR2), a cannabinoid receptor, disrupts CD8+ T and NK cell activity in the TME [29] and promotes tumor growth through the p38 MAPK pathway, making it a notable marker for HPV-positive HNSCC [30]. Increased expression of killer cell lectin like receptor K1 (KLRK1) in HPV-positive patients promotes the infiltration of CD4+ and CD8+ T cells, correlating with improved prognosis. In contrast, tumor-secreted soluble Natural killer group 2D ligands (NKG2DL) and its ligand UL16-binding protein (ULBP) 1-3 induce immune evasion, leading to poorer outcomes [31].

HPV-negative HNSCC is characterized by distinct mechanisms of immune suppression. SET and MYND domain-containing protein 3 (SMYD3) downregulates immune-related genes, including PD-L1 and type I interferons, via the DNA Methyltransferase 1 (DNMT1) pathway, resulting in decreased CD8+ T cell and macrophage infiltration [32,33]. Elevated expression of tetratricopeptide Repeat Domain 7B (TTC7B) is associated with increased macrophage infiltration, disruption of the TME, and poorer patient prognosis, suggesting its role in tumor progression [34]. Furthermore, the proteasome 26S Subunit ATPase 2 (PSMC2) gene is upregulated in HPV-negative HNSCC and promotes migratory proliferation and cell cycle progression through the PI3K/AKT/mTOR signaling pathway [35–38].

**Figure 1:**
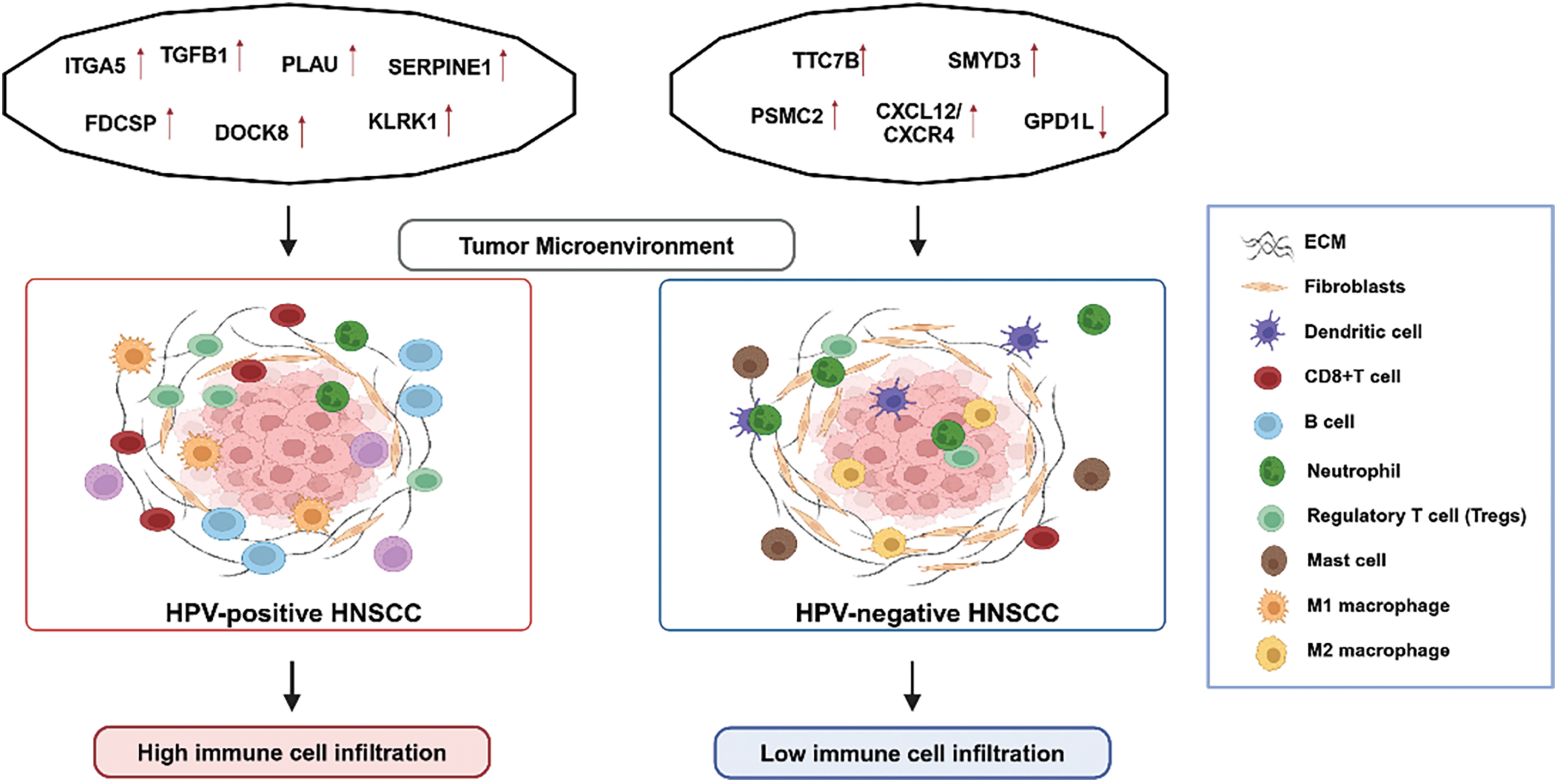
A summary of the differences in biomarkers and tumor microenvironment (TME) between HPV-positive and HPV-negative HNSCC. This figure was drawn using BioRender.

Immune infiltration within tumors is closely linked to lymph node metastasis and prognosis [39,40]. For instance, the CXCL12/CXCR4 axis drives cancer cell migration and lymph node metastasis via the ERK1/2/AP-1 signaling pathway, contributing to increased local recurrence following radiotherapy in HPV-negative cases [41,42]. In addition, the Glycerol-3-phosphate dehydrogenase 1-like (GPD1L) gene exhibits antitumor effects by modulating the hypoxia immune escape mechanism [43]. GPD1L is positively associated with eosinophils and DCs but negatively correlated with Th2 cells, Tregs, and neutrophils, reducing lymph node metastasis and inhibiting tumor spread in HPV-negative HNSCC [44]. These distinct molecular markers underscore the divergent mechanisms driving immune evasion and tumor progression in HPV-positive and HPV-negative HNSCC (Fig. 1). However, further research and validation of these molecular markers are required for their clinical use, and based on this, more precise and personalized treatment strategies can be developed.

## Immune Evasion Mechanisms in the Tumor Immune Microenvironment

The TME is a complex and dynamic and multifaceted ecosystem where cancer cells interact with various cellular and molecular components, including CAFs, tumor-associated macrophages (TAMs), tissue-associated neutrophils, and MDSCs, creating an immunosuppressive niche that supports tumor growth and immune evasion [45]. Aberrant expression of several genes regulates immune cell infiltration and contributes to cancer progression within the TME. For instance, metabolism-related genes (e.g., SMS, MTHFD2, HPRT1, DNMT1, PYGL, ADA, and P4HA1), Centrosome and spindle pole-associated protein (CSPP1), which is crucial for cytoskeletal organization and cilia formation, and Shc SH2-domain binding protein 1 (SHCBP1), which modulates cancer-related signaling pathways, collectively create an immunosuppressive TME and are linked to poor prognosis [46–49]. Therefore, strategies to modulate the TME by reducing immunosuppression and enhancing immune activation have emerged as promising approaches to improve the efficacy of immunotherapy in HNSCC.

### Cancer-associated fibroblasts (CAFs)

CAFs, a major component of the TME, influence intertumoral immune regulation and drug resistance [50]. For example, Collagen type V alpha 2 (COL5A2), secreted by CAFs, activates the PI3K/AKT pathway, leading to resistance against the epidermal growth factor receptor (EGFR) inhibitor erlotinib [51]. CAFs from chemoresistant HNSCC patients protect cancer cells by activating PI3K/AKT/p65 signaling, upregulating TGF-α, inducing the EGFR/Src/signal transducer and activator of transcription 3 (STAT3) pathway, and inhibiting apoptosis through the p53/caspase-3 pathway [52]. The PI3K/AKT signaling pathway plays a pivotal role in regulating innate immune cells, such as neutrophils, mast cells, and macrophages, thereby enhancing immune evasion [53,54]. Among the AKT isoforms, AKT3 is particularly critical, as it promotes the immunosuppressive activity of CAFs by inducing M2-like TAMs through the production of cytokines such as IL-6, IL-8, and transforming growth factor beta 1 (TGF-β1) [55]. Additionally, Fibroblast activation protein (FAP), a surface glycoprotein expressed by CAFs, is associated with immune checkpoint molecules (e.g., CTLA4, HAVCR2, and CD276) and contributes to the development and progression of HNSCC via the PI3K-AKT signaling pathway [56,57].

CAFs upregulate the secretion of cytokines, including IL-6, IL-8, IL-10, and TGF-β, from TAMs and promote immune evasion by inducing pro-tumor phenotypic changes in macrophages and neutrophils through the exclusion and depletion of CD8+ T cells [58,59]. Expression of alpha-smooth muscle actin (α-SMA), a known marker of CAFs, enables the formation of immunological synapses with Tregs in the tumor stroma, which shows anti-tumorigenic activity [60]. Aortic carboxypeptidase-like protein (ACLP) activates the CAF markers such as actin alpha 2 (ACTA2), FAP, and platelet-derived growth factor receptor beta (PDGFRB) via TGF-β1 signaling, enhancing cancer cell migration and reducing CD8+ T cell infiltration, thereby creating an immunosuppressive TME [61].

Galectins further support tumor progression by promoting cell adhesion, angiogenesis, metabolism, immune escape, and intercellular signaling. Specifically, galectin-1 aids immune evasion by inducing apoptosis in T cells, NK cells, and other immune cells [62–64]. Moreover, high expression of MHC-1 and galectin-9 in CAFs, driven by interferon (IFN) signaling, may limit CD8+ T cell-mediated anti-tumor responses in HNSCC patients [65]. These findings highlight the intricate interplay between CAFs and other TME components in driving immune evasion and tumor progression.

### Tumor-associated macrophages (TAMs)

In the TME, TAMs are pivotal in suppressing anti-cancer immune responses and facilitating immune evasion [66]. M2-like TAMs, in particular, contribute to maintaining an immunosuppressive microenvironment by inhibiting CD8+ T cells through the expression of T cell immune checkpoint ligands such as PD-L1, and secreting cytokines including CCL-2, IL-6, IL-10, and TGF-β [67]. Consequently, the polarization of TAMs toward the M2 phenotype enhances immune evasion and tumor progression.

Several factors mediate activation of M2-like TAMs. The expression of oxidized low-density lipoprotein receptor 1 (OLR1) and cerebral endothelial cell adhesion molecule (CERCAM) facilitates the infiltration of M2-polarized TAMs into the TME. NOD-like receptor pyrin domain-containing protein 3 (NLRP3) also promotes the differentiation of TAMs into the tumor-supportive M2 phenotype, a process linked to cancer progression and poor prognosis [68–70]. Notably, CERCAM, which is also highly expressed in CAFs, is associated with a high rate of M2 macrophage infiltration, with decreased infiltration of CD8+ T cells and activated NK cells [71]. In addition, reticulocalbin (RCN1), a calcium-binding protein located in the endoplasmic reticulum (ER) lumen, regulates epithelial-to-mesenchymal transition (EMT) through the AKT pathway and presumably promotes the formation of M2 TAMs by modulating the expression of markers such as CD206, Arg1, and IL-10 [72].

TAMs are closely linked to chemotherapy resistance in HNSCC. For example, EGFR overexpression upregulates key markers of M2 macrophages, including STAT6, CD163, and MRC1, which negatively affects tumor-infiltrating lymphocytes and promotes resistance to cetuximab, an EGFR-specific antibody [73]. Integrin beta 6 (ITGB6), associated with resistance to CD276 antibody therapy, facilitates immune evasion and treatment resistance by recruiting PF4+ TAMs (characterized by expression of platelet factor 4) through the CX3CL1-CX3CR1 axis, resulting in the depletion of cytotoxic CXCR6-positive CD8+ T cells [74]. Furthermore, CD276 is implicated in the migration and differentiation of M2 macrophages via the CCL2-CCR2 axis, further enhancing immune suppression and resistance to therapy [75]. TAM-secreted IL-1β mediates resistance to docetaxel (DTX) in HNSCC by upregulating intercellular adhesion molecule 1 (ICAM1), which is influenced by inflammatory cytokines [76].

### Extracellular vesicles (EVs)

Extracellular vesicles (EVs), such as exosomes derived from TAMs, function as crucial communication mediators between tumor cells and the TME. EVs not only reflect the molecular content of their parental cells but can also exhibit pro-apoptotic, immunosuppressive, or immunostimulatory properties, depending on their origin [77]. Major proteins found in EVs include CD9, CD63, and ESCRT-related components, which can serve as biomarkers for specific tumor types [78]. Chromatin-modifying protein/charged multivesicular protein (CHMP2A), a subunit of ESCRT-3, facilitates EV production by secreting CXCL10 and CXCL12, which subsequently reduce NK cell migration and inhibit NK cell cytotoxicity [79].

Exosomal thrombospondin 1 (THBS1) influences macrophage polarization toward the M1-like phenotype via p38, AKT, and stress-activated protein kinases (SAPK)/JNK signaling pathways. M1-like TAMs further enhance EMT and cancer stem cells (CSCs) characteristics through activating the Jak/STAT3 pathway [80]. EVs contribute to tumor progression by modulating the TME, increasing inflammatory cytokine levels, and inducing immunosuppression. Specifically, they suppress CD8+ T cell immune responses by downregulating protein tyrosine phosphatase non-receptor type 2 (PTPN2) expression, which accelerates tumor progression [81]. Additionally, cancer cell-derived EVs containing interferon gamma receptor 1 (IFNGR1), enhance PD-L1 expression on lymph node fibroblastic reticular cells (FRCs) through JAK1-STAT1 activation, resulting in CD8+ T cell depletion and potentially promoting lymph node metastasis of HNSCC [82].

**Figure 2:**
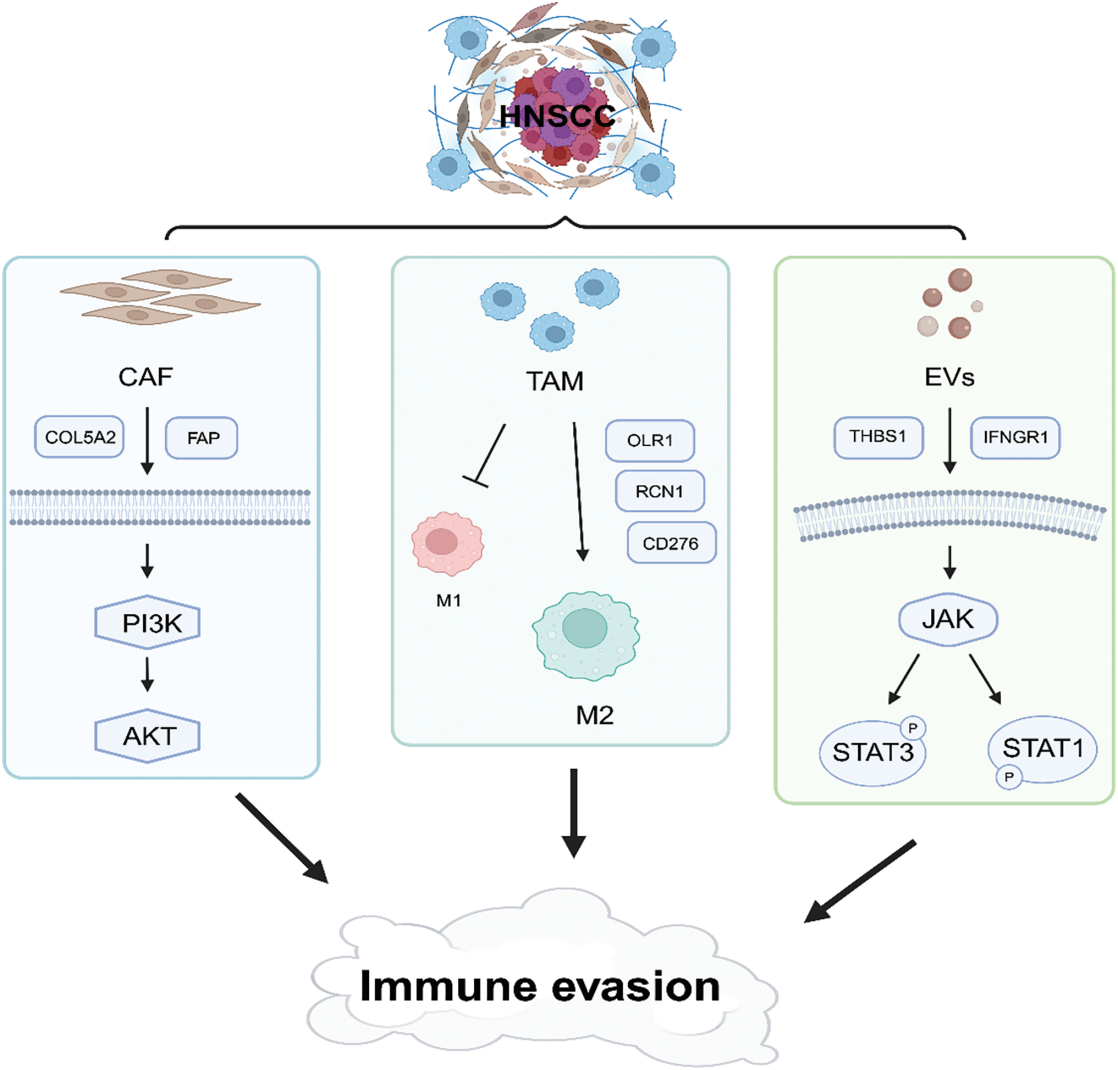
Schematic illustration of immune evasion in the tumor microenvironment. This figure was drawn using BioRender.

Previous studies have demonstrated that tumor-derived EVs remodel the TME under hypoxic conditions, thereby enhancing immune evasion, cancer cell proliferation, and angiogenesis [83,84]. Hypoxia-induced hypoxia-inducible factor 1-alpha (HIF-1α) accumulation inhibits V-type proton ATPase catalytic subunit A(ATP6V1A) expression, disrupting lysosomal homeostasis and increasing EV release [85]. Hypoxia-driven exosomes exhibit elevated expression of zinc Finger E-Box Binding Homeobox 1 (ZEB1), which induces TAM phenotype switching through STAT3 signaling, thereby amplifying immunosuppressive activity [86]. Moreover, EVs contain abundant microRNAs (miRNAs), including miR-21-5p, which promotes angiogenesis in HNSCC through the HIF-1α pathway [87]. These findings underscore the multifaceted roles of EVs in reshaping the TME, promoting immune evasion, and driving cancer progression and metastasis in HNSCC. Targeting EV-mediated pathways represents a promising avenue for therapeutic intervention in HNSCC (Fig. 2).

## Evasion of Cancer Immune Surveillance

### PD-1/PD-L1

PD-L1 expression can be upregulated by oncogenic transcription factors, such as MYC, AP-1, and STAT, through direct promoter binding or activation of pathways, including MAPK, PTEN/PI3K/AKT, and EGFR. Additionally, DNA double-strand breaks can trigger STAT activation via the ATM/ATR/Chk1 kinase axis, further regulating PD-L1 expression [88–90]. Tumor-associated PD-L1 is primarily induced by tumor-infiltrating lymphocyte (TIL)-derived interferon gamma (IFN-γ), which promotes apoptosis and dysfunction of activated T-cells, thereby attenuating the immune response and facilitating tumor growth [91].

High expression of oxidized low-density lipoprotein receptor 1 (OLR1) is associated with recruitment of immune cells such as macrophages and cancer-associated fibroblasts. OLR1 activates the PD-1/PD-L1 axis, Toll-like receptor signaling, STAT3 pathway. OLR1 also upregulates the stem cell marker CD44, contributing to immune surveillance evasion and malignant cell proliferation [92]. Similarly, serine hydroxymethyltransferase 2 (SHMT2), highly expressed in HNSCC, inhibits CD8+ T-cell infiltration and promotes immune escape by regulating the MIF/CD44 axis. SHMT2 is associated with poor prognosis and increased PD-L1 expression [93]. Toll-like receptor 9 (TLR9), another key player in immune evasion, upregulates PD-L1 and promotes tumor growth through the PARP1/STAT3 pathway, enhancing tumor cell proliferation, migration, and invasion [94].

In HNSCC, EMT induced by TGF-β signaling mediates immunosuppression and activation of immune checkpoints, such as PD-1, PD-L1, and CTLA-4, particularly in cells with mesenchymal phenotypes. This leads to enhanced tumor growth and metastasis [95]. The PD-1 immune checkpoint interacts with two ligands: PD-L1 and PD-L2. While PD-L1 is primarily induced by IFN-γ, PD-L2 is equally responsive to IFN-β and IFN-γ, regulated by interferon regulatory factor 1 (IRF1) and STAT3 [96]. Notably, glycosylation of PD-L2 by fucosyltransferase 8 (FUT8) stabilizes its expression and enhances EGFR/STAT3 signaling. This glycosylated PD-L2 promotes immune evasion via PD-1 binding, leading to T-cell dysfunction and allowing cancer cells to escape immune surveillance [97]. These mechanisms illustrate the complexity of immune checkpoint pathways in HNSCC, highlighting their critical role in tumor progression and resistance to immune surveillance.

Recent clinical trials targeting these pathways further underscore the ongoing efforts to improve immunotherapy for HNSCC. In a phase II study (NCT03264066) of patients with HNSCC, the combination of cobimetinib (an MEK inhibitor) and atezolizumab (an anti-PD-L1 monoclonal antibody) resulted in a 20% response rate [4]. A randomised phase III study (NCT02369874) in patients with recurrent or metastatic HNSCC reported an objective response rate of 17.9% with durvalumab (anti-PD-L1) and 18.2% with durvalumab plus tremelimumab (anti-CTLA-4) [98]. Another randomised phase III study (NCT02551159) in patients with PD-L1-high HNSCC showed an impressive response rate of 16% for durvalumab alone and 48% for the combination with tremelimumab [99]. Cadonilimab a novel bispecific antibody that targets both PD-1 and CTLA-4, showed an 18.2% response rate in a phase 1b/2 study (NCT03852251), but its efficacy in HNSCC needs further validation [100].

### Human leukocyte antigen/major histocompatibility complex (HLA/MHC)

MHC class I downregulation is frequently observed in various tumors and represents a crucial mechanism of immune evasion, as it impairs the recognition and activation of CD8+ cytotoxic T cells [101]. In HNSCC, decreased expression of gamma subunit-4 (GNG4), which is involved in the PI3K/AKT and NF-κB pathways, inhibits the expression of MHC class 1 independently of the IFN-γ signaling, contributing to resistance to PD-1 blockade [102]. In addition, increased expression of HIF1A-AS2, a hypoxia-regulated long non-coding RNA (lncRNA), is associated with elevated HIF-1α levels, leading to enhanced autophagic degradation of MHC class I and reduced CD8+ tumor-infiltrating lymphocytes, ultimately weakening the immune response against tumors [103].

In contrast, HNSCC patients with elevated expression of LINC02195, another lncRNA, exhibit increased expression of MHC class I, which positively correlates with CD8+ and CD4+ T cell infiltration [104]. Targeting Flap endonuclease 1 (FEN1) has been shown to activate the DNA damage response by downregulating STAT1/STAT2, thereby increasing HLA/MHC class I expression and reducing PD-L1 levels. This process overcomes FEN1-mediated immunosuppression, promoting CD8+ T cell-mediated anti-tumor activity through cell cycle arrest and apoptosis of tumor cells [105,106].

Inhibition of fibroblast growth factor receptor 1 (FGFR1) increases the expression of MHC class I and II, improving antigen presentation by increasing human leukocyte antigen (HLA) expression through MAPK pathway inhibition [107]. While most benign tumor cells express HLA, its expression on malignant cells can be induced by interferon and is typically colocalized with T cells within the tumor parenchyma [108]. Conversely, Homeobox B7 (HOXB7) suppresses the IFN-γ/STAT1/class II major histocompatibility complex transactivator (CIITA) axis and downregulates HLA class II expression through the MAPK pathway, thereby facilitating immune evasion [109]. CD3D, a component of the T-cell receptor (TCR)/CD3 complex, has demonstrated a better response to immunotherapy in HNSCC patients. Its upregulation is associated with increased immune cell infiltration, elevated expression of HLA-related genes, and enhanced activation of the JAK/STAT signaling pathway, highlighting its potential as a therapeutic target to bolster immune-mediated tumor clearance [110].

### Molecular approaches to immune evasion in tumors

IFN-γ plays a critical role in T cell-mediated antitumor immune responses by modulating tumor-killing activity and thereby influencing tumor immune evasion [111]. IFN-γ-induced SP140 expression in tumors inhibits STAT1 while promoting infiltration of M1 macrophages and CD8+ T cells [112]. Although STAT1 functions as a tumor suppressor, its activation in TAMs upregulates inducible nitric oxide synthase (iNOS) and arginase 1, resulting in T cell suppression [113]. Interestingly, deficiency of the N6-methyladenosine (m6A) reader YTH N6-methyladenosine RNA binding protein 2(YTHDF2) reprograms TAMs into an antitumor phenotype by targeting the IFN-γ/STAT1 signaling axis, thereby enhancing CD8+ T cell-mediated immunity [114].

While immunomodulatory strategies typically focus on bolstering tumor-infiltrating CD8+ T cell activity, CD4+ T cells also significantly influence the TME by secreting cytokines that enhance CD8+ T cell infiltration [115]. In HPV-positive tumor cells, induction of CXCL13 in CD4+ T cells lead to increased secretion of IFN-γ, which contributes to elevated tumor-infiltrating lymphocyte (TIL) counts and improved immune cell infiltration, thereby enhancing the prognosis of HNSCC patients [116,117]. Increased CXCL13 levels also facilitate B cell recruitment and drive the formation of tertiary lymphoid structures (TLS) [116,118]. Unlike encapsulated secondary lymphoid organs (SLOs), TLSs allow free movement of immune cells, enabling localized T cell recognition of tumor-associated antigens with the assistance of B cells, thereby intensifying the local immune responses at the site of TLS formation [119].

The membrane-bound glycoprotein semaphorin-4A (SEMA4A), crucial for T cell co-stimulation and a key driver of Th 2 responses, plays an important role in generating immune aggregates through interactions between tumor-infiltrating B cells (TIL-Bs), endothelial cells, and T cells [120]. In HNSCC, IL17A expression, which is implicated in TLS formation, is positively correlated with increased infiltration of T and B cells, suggesting its critical role in immune regulation [121].

Toll-like receptors (TLRs), key pattern recognition receptors in the innate immune response, are expressed on a range of immune cells, including TAMs [122]. TLR10, by competing with other costimulatory TLRs, activates the PI3K/AKT to produce IL-1Ra, a tumor suppressor [123]. Expression of TLR10 is also linked to early B cell development. Activation of the TLR4 pathway generates reactive oxygen species (ROS) and drives NF-κB nuclear translocation in neutrophils [124]. Dual inhibition of interleukin-1 receptor-associated kinases (IRAK-4 and IRAK-1), downstream mediators of TLR signaling, suppresses the expression of anti-apoptotic proteins such as Bcl-2 and Bcl-xL in chemoresistant HNSCC. This inhibition also reduces the secretion of the pro-inflammatory cytokine IL-6, mitigating its role in shaping an immunosuppressive TME [125].

### Latest immunotherapies and research trends for HNSCC treatment

Recent clinical trials and studies suggest that immune checkpoint inhibitors, including nivolumab (anti-PD-1) and ipilimumab (anti-CTLA-4), demonstrate potential in overcoming immune evasion in HNSCC (NCT02741570) [126]. Furthermore, the combination of the vascular endothelial growth factor receptor (VEGFR) inhibitor apatinib and the anti-PD-1 antibody camrelizumab has demonstrated a pathological response rate of approximately 40%, underlining the potential of immunomodulatory treatments (NCT04393506) [127]. Other novel immunotherapeutic strategies include the combination of cetuximab with a TLR8 agonist, which enhances anti-tumor immune responses by regulating the TIGIT signaling pathway (NCT02124850) [128]. Furthermore, urelumab, a CD137 agonist, has demonstrated the capacity to activate immune-related pathways when combined with nivolumab, suggesting the potential to enhance immune responses in HNSCC (NCT02253992) [129].

The dysregulation of the PI3K/AKT/mTOR pathway in tumor cells plays a pivotal role in immune evasion and tumor progression, leading to immune cell exhaustion and suppression [21,130]. In response to this, ongoing clinical trials are evaluating the PI3K inhibitor buparlisib (NCT01527877), with studies investigating its combination with cetuximab to enhance treatment efficacy in recurrent and metastatic HNSCC [131]. Additionally, emerging strategies targeting immune checkpoint molecules offer new therapeutic potential. The combination of eftilagimod alpha (a soluble LAG3 protein) and pembrolizumab has demonstrated tumor regression and immune activation, particularly in tumors with poor T-cell infiltration (NCT03625323) [132].

Meanwhile, BL-B01D1, a bispecific antibody-drug conjugate targeting EGFR and HER3, has exhibited an objective response rate of approximately 34% in a phase 1 clinical trial for HNSCC (NCT05194982) [133]. In addition, nintedanib is currently being evaluated in a phase 2 trial for patients with FGFR mutations (NCT03292250) [134]. These advancements underscore the continuous progress in HNSCC treatment and the growing potential of combination immunotherapies.

## Conclusion

The intricate interplay between molecular mechanisms, the TME, and immune evasion underscores the challenges in effectively treating HNSCC. HPV status significantly influences the immune landscape and prognosis. The distinct immune evasion strategies employed by HPV-positive and HPV-negative tumors highlight the critical need for personalized therapeutic approaches tailored to the specific characteristics of each tumor type. Recent advances in the understanding of biomarkers, tumor-associated immune cells, and molecular pathways have facilitated the identification of potential targets to counteract immunosuppression and enhance the efficacy of immunotherapy. Leveraging these insights in future research and clinical strategies holds promise for reshaping the treatment paradigm, offering improved outcomes and prolonged survival for patients with HNSCC.

## Data Availability

Data sharing is not applicable to this article as no datasets were generated or analyzed during the current study.
